# Randomised Controlled Double-Blind Non-Inferiority Trial of Two Antivenoms for Saw-Scaled or Carpet Viper (*Echis ocellatus*) Envenoming in Nigeria

**DOI:** 10.1371/journal.pntd.0000767

**Published:** 2010-07-27

**Authors:** Isa S. Abubakar, Saidu B. Abubakar, Abdulrazaq G. Habib, Abdulsalam Nasidi, Nandul Durfa, Peter O. Yusuf, Solomon Larnyang, John Garnvwa, Elijah Sokomba, Lateef Salako, R. David G Theakston, Ed Juszczak, Nicola Alder, David A. Warrell, for the Nigeria-UK EchiTab Study Group

**Affiliations:** 1 Department of Community Medicine, Bayero University of Kano, Kano, Nigeria; 2 General Hospital Kaltungo, Kaltungo, Gombe State, Nigeria; 3 Department of Medicine, Bayero University Kano, Kano, Nigeria; 4 Special Projects Unit, Federal Ministry of Health, Abuja, Nigeria; 5 Faculty of Veterinary Medicine, Ahmadu Bello University, Zaria, Nigeria; 6 Alistair Reid Venom Research Unit, Liverpool School of Tropical Medicine, Liverpool, United Kingdom; 7 Department of Pharmacology, University of Jos, Jos, Nigeria; 8 Department of Pharmacology and Therapeutics, University of Ibadan, Ibadan, Nigeria; 9 Centre for Statistics in Medicine, University of Oxford, Oxford, United Kingdom; 10 Nuffield Department of Clinical Medicine, John Radcliffe Hospital, University of Oxford, Oxford, United Kingdom; Liverpool School of Tropical Medicine, United Kingdom

## Abstract

**Background:**

In West Africa, envenoming by saw-scaled or carpet vipers (*Echis ocellatus*) causes great morbidity and mortality, but there is a crisis in supply of effective and affordable antivenom (ISRCTN01257358).

**Methods:**

In a randomised, double-blind, controlled, non-inferiority trial, “EchiTAb Plus-ICP” (ET-Plus) equine antivenom made by Instituto Clodomiro Picado was compared to “EchiTAb G” (ET-G) ovine antivenom made by MicroPharm, which is the standard of care in Nigeria and was developed from the original EchiTAb-Fab introduced in 1998. Both are caprylic acid purified whole IgG antivenoms. ET-G is monospecific for *Echis ocellatus* antivenom (initial dose 1 vial) and ET-Plus is polyspecific for *E. ocellatus, Naja nigricollis* and *Bitis arietans* (initial dose 3 vials). Both had been screened by pre-clinical and preliminary clinical dose-finding and safety studies. Patients who presented with incoagulable blood, indicative of systemic envenoming by *E. ocellatus*, were recruited in Kaltungo, north-eastern Nigeria. Those eligible and consenting were randomly allocated with equal probability to receive ET-Plus or ET-G. The primary outcome was permanent restoration of blood coagulability 6 hours after the start of treatment, assessed by a simple whole blood clotting test repeated 6, 12, 18, 24 and 48 hr after treatment. Secondary (safety) outcomes were the incidences of anaphylactic, pyrogenic and late serum sickness-type antivenom reactions.

**Findings:**

Initial doses permanently restored blood coagulability at 6 hours in 161/194 (83.0%) of ET-Plus and 156/206 (75.7%) of ET-G treated patients (Relative Risk [RR] 1.10 one-sided 95% CI lower limit 1.01; P = 0.05). ET-Plus caused early reactions on more occasions than did ET-G [50/194 (25.8%) and 39/206 (18.9%) respectively RR (1.36 one-sided 95% CI 1.86 upper limit; P = 0.06). These reactions were classified as severe in 21 (10.8%) and 11 (5.3%) of patients, respectively.

**Conclusion:**

At these doses, ET-Plus was slightly more effective but ET-G was slightly safer. Both are recommended for treating *E. ocellatus* envenoming in Nigeria.

**Trial Registration:**

Current Controlled Trials ISRCTN01257358

## Author Summary

Snake bite threatens millions of poor rural folk throughout Africa. In Nigeria, as in many countries of sub-Saharan Africa, it takes a terrible toll on human life and limb. Over the years, the news for those exposed to snake bite has been generally bad: withdrawal of antivenom manufacturers, increasing cost and, most recently, the marketing of ineffective or fake antivenoms in the region. Our paper reports encouraging results achieved by two antivenoms created as a direct consequence of the present crisis in antivenom supply for Africa. They have been assessed in the most powerful trial ever attempted in this field. The trial showed that in people with non-clotting blood following carpet viper bite, the commonest cause of snake bite morbidity and mortality in the West African savannah, administration of the antivenoms- EchiTAb G and EchiTAb Plus-ICP led to permanent restoration of blood clotting in 76% and 83% of the patients within 6 hours, respectively. Generally mild early adverse reactions were recorded in 19% and 26%, respectively. Both antivenoms proved effective and acceptably safe and can be recommended for treating carpet viper envenoming in Nigeria.

## Introduction

Bites by saw-scaled or carpet vipers (*Echis ocellatus*) are frequent in the savanna region of West Africa, where agricultural workers and their children are at greatest risk [1]–[6]. Systemic haemorrhage, consumption coagulopathy, shock and debilitating local tissue necrosis may ensue [6]. Fatalities result from haemostatic failure susceptibility to which is conveniently detected and monitored by the 20 minute whole blood clotting test [6] which correlates with a plasma fibrinogen concentration of about 0.5 g/l [7] and has been used in several previous antivenom trials [3]–[6], [8]–[17]. In Nigeria, where untreated case fatality exceeds 10–20%, *E. ocellatus* causes hundreds of deaths each year [1], [18], [19]. In recent years, antivenom has become scarce, costly and inaccessible to most patients [18], [20]–[23]. This provides an entrée for unscrupulous marketing of geographically-inappropriate products that can prove clinically disastrous [3], [24], [25]. Improving the treatment of snake bite victims in Nigeria demands solutions to economic, logistical, marketing, distribution and storage problems associated with antivenom supply and provision of better training for medical personnel to optimize antivenom use [26]–[28]. The development of safe, effective and affordable antivenoms is a priority [28]. In the 1990s, the Federal Ministry of Health in Nigeria (FMHN) supported the development of a new ovine Fab monospecific antivenom raised against Nigerian *E. ocellatus* venom (EchiTAb-Fab antivenom) by MicroPharm, UK. This antivenom was tested [8], [29], registered by the Nigerian National Agency for Food and Drug Administration and Control (NAFDAC) and used in Nigeria from 1998–2000. However, its use, like that of CroFab*®* in the United States [30], was complicated by recurrent envenoming [31] attributable to rapid clearance of the Fab fragments [8]. To overcome this problem, it was replaced by a caprylic acid-refined, whole IgG antivenom (EchiTAb-G) (ET-G) with the same specificity. This proved clinically effective during compassionate clinical release in Nigeria, becoming the standard of care. During pre-trial use at Kaltungo, in early 2005, 146 of 182 (80%; 95% confidence interval 74%–85%) patients envenomed by *E. ocellatus* showed permanent restoration of blood coagulability 6 hours after an initial dose of 1 vial of ET-G (unpublished data). This antivenom was registered by NAFDAC (registration number A6-0078). Recently, a new equine whole IgG antivenom (EchiTAb-Plus-ICP) (ET-Plus) was prepared by Instituto Clodomiro Picado, Costa Rica, also refined using caprylic acid [32]–[34]. It was raised against venoms of *E. ocellatus*, puff adder (*Bitis arietans*) and black-necked spitting cobra (*Naja nigricollis*), the taxa of greatest medical importance in Nigeria. Pre-clinical (rodent ED_50_ assay) and preliminary clinical dose-finding and safety studies suggested that initial doses of 1 vial of ET-G and 3 vials of ET-Plus might cure the coagulopathy in at least two thirds of patients [35].

A natural progression of this work was a larger Phase III head-to-head comparison of both antivenoms in a non-inferiority design, since ET-G was the established standard of care. Since the untreated case fatality rate for *E. ocellatus* envenoming, inferred from results of treatment with inappropriate non-specific antivenoms, has been reported as 12.1% (95% CI: 6.3–22.1%) [3] and 15.8% (95% CI: 10.4–23.4%) [1], it was considered unethical to include a placebo comparator arm. In this paper, we compare the effectiveness in correcting coagulopathy and safety of ET-Plus with those of ET-G (standard treatment) for envenoming by *E. ocellatus*, in a randomised controlled double-blind non-inferiority trial.

## Methods

The protocol for this trial and supporting CONSORT checklist are available as supporting information; see Protocol S1 and Checklist S1.

### Objectives

In the face of the current crisis in antivenom availability in Nigeria, our main aim was to evaluate effective and acceptably safe treatments for *E. ocellatus* envenoming in a randomised controlled double-blind non-inferiority trial, comparing ET-Plus, a new antivenom, with ET-G, an antivenom of established effectiveness which has been the standard for care in Nigeria since 2005.

### Participants

#### Patient eligibility

All patients presenting to Kaltungo General Hospital, Gombe State, Nigeria with a history of snake bite were assessed for their eligibility.

Inclusions - all patients of any age provided that:

1. they had incoagulable blood as defined by the 20 minute whole blood clotting test (20WBCT) [6], [7] indicative of systemic envenoming by *E. ocellatus* in this geographical area [1], [6], [8], [9], [11].
2. they had been bitten within the previous 72 hours
3. they or their relatives gave informed consent to admission, treatment and investigation

Exclusions:

1. patients who had already received antivenom for their present snake bite
2. pregnant women (as required by the local ethics committee)
3. patients whose signs and symptoms of intracerebral haemorrhage (coma, and/or lateralising neurological signs) demanded immediate treatment with an antivenom of established clinical effectiveness (see below).
4. patients with a severe unrelated medical condition such as advanced AIDS or tuberculosis (as required by the local ethics committee)

Ineligible patients were treated, outside the trial, with a large initial dose of ET-G antivenom, currently standard of care in Nigeria, or South African Vaccine Producers (SAVP) Echis antivenom [6], [9].

Medical history and results of physical examination were recorded on standard forms on admission and at least daily thereafter until discharge.

### Interventions

#### Antivenom

Both antivenoms had passed their manufacturers'standard tests for neutralising potency, purity, lack of pyrogenicity (endotoxin levels equal to or less than 6 eu/ml) aggregate content (3.0±0.4% by gel filtration chromatography), high molecular mass components (molecular masses above that of IgG <5% by SDS-PAGE), appropriate protein concentration (ET-Plus 40 mg/ml, ET-G, 37 mg/ml), lack of abnormal toxicity, sterility, pH (ET-Plus 6.83, ET-G 5.8-6.2) and other recommended tests [33]–[35]. The batches of antivenom used in the study were: ET-Plus 3710204LQ (exp. February 2007) and 4260308PALQ (exp. March 2011); ET-G EoG00068 (exp. January 2007), EoG00077 (exp. May 2007), EoG00084 (exp. July 2007).

The initial doses of antivenom chosen for this study were derived by pre-clinical and preliminary clinical dose-finding/safety studies as follows [35]. Median effective doses (ED_50_) of candidate antivenoms, obtained by using standard neutralisation assays in rodents, were the basis for calculating doses capable of neutralising the maximum amount of venom that could be milked from captive *E. ocellatus*. These doses were tested clinically in volunteer patients envenomed by *E. ocellatus* in a modified 3+3 dose escalation protocol. The resulting “minimum effective safe doses” were 3 vials of ET-Plus and 1 vial of ET-G [35]. Antivenoms were administered intravenously at a rate of approximately 2 ml/minute. The initial dose was repeated at 6 hourly intervals until blood coagulability, assessed by 20WBCT, was restored permanently or until the case was deemed a “treatment failure”, defined as failure to achieve permanent restoration of blood coagulability within 24 hours of the first dose. In such cases, “rescue treatment” was given: 2 vials of ET-G (currently the standard of care) or, when available, SAVP Echis antivenom [6], [9]. To exclude recurrent envenoming, 20WBCT was repeated 6 hourly for 48 hours after blood coagulability had been restored and daily thereafter up to 96 hours after the start of antivenom treatment to cover the longest reported delay in recurrence [6], [8].

#### Other treatment

Pain was treated with oral paracetamol (adult 1 g 6 hourly) or codeine phosphate (30 mg). Fresh compatible HIV, HBV, HCV-negative whole blood (screened through Columbia University's International Centre for AIDS Care & Treatment Program - ICAP) was transfused if the haematocrit fell below 20%. Envenomed limbs were nursed in the most comfortable position. A booster dose of tetanus toxoid was given to every patient. If local necrosis developed, the affected area was surgically débrided and broad-spectrum antimicrobial cover (flucloxacillin, gentamicin and metronidazole) was started immediately.

### Outcomes

#### Primary outcome

The primary outcome was permanent restoration of blood coagulability, judged by 20WBCT at 6 hours after initiation of antivenom treatment. “Permanent” implied restoration after which there was no (further) recurrence of blood incoagulability. This was assessed by repeating the 20WBCT 6, 12, 18, 24 and 48 hr after the initial dose of antivenom.

Restoration of blood coagulability, assessed by 20WBCT, was taken as evidence of the effectiveness of the antivenom in neutralising anti-haemostatic effects of *E. ocellatus* venom. Persistence of incoagulable blood six hours after the first or subsequent doses of antivenom was the indication for repeating the initial dose of antivenom as in previous studies [6], [8], [9], [11].

#### 20WBCT method

2ml of venous blood was taken into a 26×6 mm new, clean, dry glass test tube, left upright and undisturbed for 20 minutes at ambient temperature and then tipped once. If the blood poured out in liquid form, the result was termed “unclotted”; otherwise, the result was termed “clotted”. If there was any doubt about the result, the test was repeated immediately, using as control blood from a healthy person, usually the patient's relative [1], [6], [7], [8], [9], [11].

#### Secondary (safety) outcomes

Secondary (safety) outcomes included the incidences of (1) early anaphylactic-like, (2) pyrogenic, and (3) late serum sickness type antivenom reactions. Patients were observed closely by the clinician for any symptoms or signs of early antivenom reactions during and for a period of 4 hours after antivenom administration.

Early reactions were classified as:

1. Anaphylactic-like
  - mild - pruritus and/or urticaria only
  - severe - gastrointestinal symptoms (vomiting, diarrhoea, colicky abdominal pain), bronchospasm, or fall in systolic blood pressure below 90 mmHg.
  Anaphylactic-like reactions were treated by temporarily stopping antivenom injection and giving 0.1% adrenaline by intramuscular injection (adult dose 0.5–1 ml; children 0.01 ml/kg body weight), followed by chlorphenamine maleate by slow intravenous injection (adult dose 10 mg; children 0.2 mg/kg body weight) and hydrocortisone hemisuccinate by slow intravenous injection (adult dose 100 mg; children 2 mg/kg body weight). After the symptoms of the reaction had subsided, administration of the full dose of antivenom was completed.
2. Pyrogenic- increase in oral temperature to 38° or above with or without rigors. They were treated by temporarily stopping antivenom injection and using physical cooling (tepid sponging and fanning). After the fever and other symptoms had subsided, administration of the full dose of antivenom was completed.
3. Late serum sickness-type reactions. Patients were encouraged to return to hospital 2 weeks after the bite to allow assessment of symptoms and signs of serum sickness which was treated with chlorphenamine or prednisolone tablets.

#### Ancillary clinical outcomes

On admission, history and physical examination were recorded on standard proformas. Sites of external spontaneous systemic bleeding (gums, nose etc.) were examined frequently until the bleeding had ceased permanently. Subsequently, patients were questioned about new symptoms and were examined at regular intervals, at least twice daily, with special emphasis on vital signs, evidence of recurrent envenoming and evolution of local envenoming. Duration of hospital stay was measured to the nearest day. Patients were discharged from hospital by the clinician who was masked to the particular antivenom that they had received. Haematocrit (packed cell volume - PCV) was checked at discharge.

Patients were asked to attend for follow-up assessment 2 weeks after the bite for physical examination with particular attention to local envenoming and serum sickness (see above).

### Sample size

The trial was designed to demonstrate non-inferiority of ET-Plus compared to ET-G. A 10% non-inferiority margin was deemed acceptable (i.e. we stipulated that at least 70% of ET-Plus patients must have permanent restoration of blood coagulability at 6 hours for it to be deemed non-inferior to ET-G). A sample size of 198 in each group provides 80% power to detect this non-inferiority margin difference of 10%, at a 5% one-sided significance level. It was anticipated that there would be no losses to follow up.

### Randomisation and masking

Patients were screened for eligibility and enrolled by one of the study clinicians who then contacted the hospital pharmacist directly for randomisation and provision of the allotted antivenom. Patients were allocated to receive either ET-Plus or ET-G with equal probability (allocation ratio 1∶1) using simple randomisation. The random number sequence was generated using a table of random numbers. Treatment allocations were concealed by using sequentially numbered opaque sealed envelopes held by the hospital pharmacist who was otherwise independent of the study. He provided the masked antivenoms after reconstituting them to total volumes of 40 mls with sterile water for injection in an unmarked syringe. Diluted in this way, ET-Plus 3∶4, ET-G 1∶4, both antivenoms appeared as identical colourless solutions. The patient, ward staff and treating clinicians, who also assessed the outcome, were thus masked to the identity of the particular antivenom used.

### Statistical methods

Patients were analysed in the groups to which they were assigned regardless of deviation from the protocol or treatment received (intention-to-treat population). Baseline demographic factors and clinical characteristics were summarised using counts (percentages) for categorical variables, mean (standard deviation [SD]) for normally distributed continuous variables, or median (interquartile [IQR] or entire range) for other continuous variables.

To determine the magnitude and direction of the treatment effects for dichotomous outcomes, relative risks and one-sided 95% confidence intervals were calculated; the lower limit was provided when comparing antivenom effectiveness (where an increase in positive events is desirable) whereas the upper limit was provided when comparing safety (where a decrease in negative events is desirable). Continuous outcomes were checked for normality and the treatment groups were compared using either the *t* test (for normally distributed data) or the Mann Whitney-U test (for non-normally distributed data). Treatment effects were presented as a corresponding difference in means or medians (plus one-sided 95% confidence intervals).

### Ethics

ET-G and ET-Plus were given limited registration for clinical trials by NAFDAC. The trial was sanctioned by NAFDAC and the Gombe State Medical Research Ethics Committee. Written informed consent (in English or Hausa, the common language of this region) was obtained from the patients after they had read the information sheet and discussed it with medical staff. Oral explanations in Tangale or Fulani were also available.

## Results

### Recruitment and participant flow

**Figure 1:**
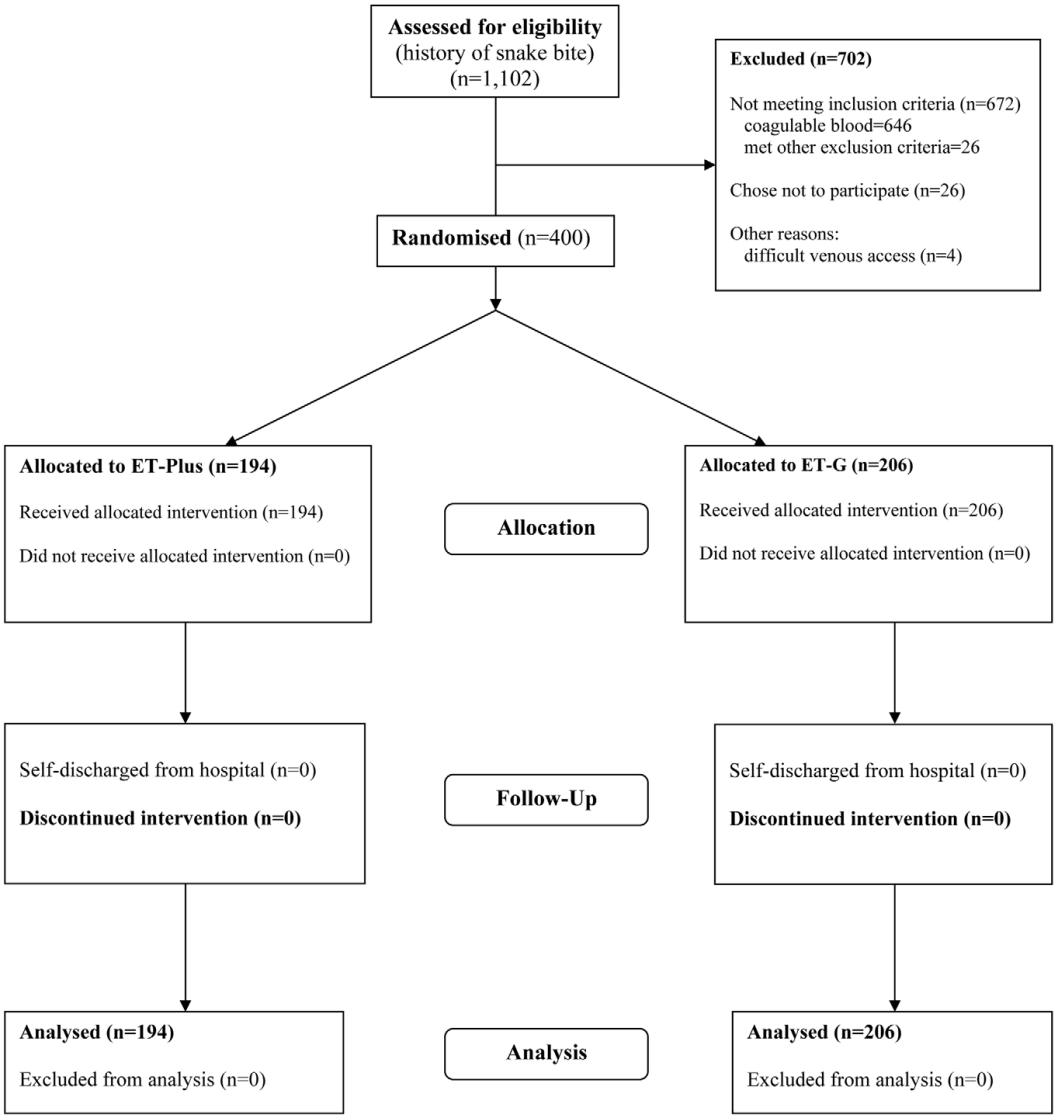
Flow of participants through the trial.

The trial was conducted during the period 2005–2007, but recruitment was possible for only 9 months, because of staffing problems and delayed importation and authorisation by NAFDAC of the second consignment of ET-Plus antivenom. During this time, 1102 patients presented to the hospital with a history of snake bite and were assessed for eligibility (Figure 1). Among 646 patients who were ineligible because their blood was coagulable, 74 had been bitten by snake species other than *E. ocellatus* [night adders (*Causus maculatus*), burrowing asps (*Atractaspis dahomeyensis* and *A. watsoni*) and cobras (*Naja nigricollis*) identified by examination of the dead snakes brought by these patients] and 572 showed only local or no envenoming. Among 456 with incoagulable blood (20WBCT), indicating systemic envenoming by *E. ocellatus*, 26 refused to join the study, 4 were excluded because of difficult venous access and 26 were ineligible for the following reasons as per protocol: pregnancy (10), antivenom treatment for their present bite (2), evidence of coma/cerebral haemorrhage on admission (4), severe underlying illness (HIV/AIDS) (2), bitten more than 72 hr previously (8). Of 400 recruited to the study, 68 were children under the age of 14.

### Baseline characteristics

One hundred and ninety four patients allocated ET-Plus proved to be broadly similar to 206 allocated ET-G in their demographic and clinical characteristics, including age, height and weight, proportion who brought the dead snake (*Echis ocellatus*) that had bitten them, sex, proportion of children, delay between bite and admission, site of bite, severity of local envenoming and incidence of spontaneous systemic bleeding (Table 1).

**Table 1:** Baseline demographic and clinical characteristics (intention-to-treat population).

Characteristic	EchiTAb Plus-ICP	EchiTAb G
	(n = 194)	(n = 206)
**Age (yrs) - mean (SD)**	25.4 (12.0)	25.7 (13.1)
**[range]**	[6–60]	[7–60]
**aged <14 yrs**	29 (14.9%)	39 (18.9%)
**Male**	138 (71.1%)	137 (66.5%)
**Weight (Kg)**		
**Mean (SD)**	52.1 (14.0)	52.3 (15.4)
**Median (range)**	54.0 (14.0–86.7)	55.0 (19.0–92.5)
**Missing**	0	2 (1.0%)
**Height (m)**		
**Mean (SD)**	1.59 (0.16)	1.57 (0.16)
**Median (range)**	1.60 (1.09–1.90)	1.60 (1.08–1.88)
**Missing**	0	1 (0.5%)
**Dead ** ***Echis ocellatus*** ** brought**	83 (42.8%)	77 (37.4%)
**Missing**	111 (57.2%)	129 (62.6%)
**Occupation**		
** farmers**	49 (25.3%)	69 (33.5%)
** cattle rearers**	26 (13.4%)	27 (13.1%)
** other**	119 (61.1%)	110 (53.4%)
**Circumstance of bite**		
** working**	27 (14.3%)	29 (14.2%)
** walking**	64 (33.9%)	84 (41.2%)
** other**	98 (51.9%)	91 (44.6%)
** Missing**	5 (2.6%)	2 (1.0%)
**Time from bite to admission (hours)Median**	8.7	9.3
** (IQR)**	(4.3–17.5)	(4.7–16.6)
** (entire range)**	(0.20–70.32)	(0.10–72.00)
**Site of bite**		
** lower limb**	143 (73.7%)	155 (75.2%)
** upper limb**	49 (25.3%)	48 (23.3%)
** other**	2 (1.0%)	3 (1.5%)
**Local presenting features**		
** any swelling**	193 (99.5%)	206 (100.0%)
** severe swelling** *	113 (58.2%)	130 (63.1%)
** blistering**	11 (5.7%)	11 (5.3%)
** local bleeding**	126 (64.9%)	139 (67.5%)
** painful regional lymph nodes**	124 (63.9%)	148 (71.8%)
** bruising**	2 (1.0%)	2 (1.0%)
**Systemic presenting features**		
** vomiting**	63 (32.5%)	66 (32.0%)
** bleeding gums**	41 (21.1%)	49 (23.8%)
** epistaxis**	9 (4.6%)	5 (2.4%)
** haematuria**	7 (3.6%)	12 (5.8%)
** haematemesis**	7 (3.6%)	10 (4.9%)
** haemoptysis**	4 (2.1%)	0
** melaena**	3 (1.6%)	3 (1.5%)
** Spontaneous systemic bleeding**	71 (36.6%)	79 (38.3%)
** shock/low BP (systolic <90 mmHg)**	10 (5.2%)	16 (7.8%)

SD – standard deviation.

IQR- interquartile range (25^th^ - 75^th^ percentile).

*Severe swelling  =  swelling affecting more than half of the limb.

All patients complained of pain; none was in shock or had an un-recordable blood pressure.

### Outcomes

#### Primary end point

Coagulopathy response (judged by 20WBCT) - blood coagulability was permanently restored within six hours of the initial dose in 161 of 194 (83.0%) patients given three vials of ET-Plus and in 156 of the 206 (75.7%) patients given one vial of ET-G (Table 2; RR 1.10 one-sided 95% CI lower limit 1.01; P = 0.05).

**Table 2:** Summary of primary and secondary (safety) outcomes plus comparative statistics (intention-to-treat population).

Outcome	EchiTAb Plus-ICP	EchiTAb G	Relative Risk^1^	P-value^2^
	(n = 194)	(n = 206)	(one-sided 95% CI)	
**PRIMARY OUTCOME**				
**Permanent restoration of blood coagulability (20WBCT) 6 hr after 1^st^ dose of antivenom**	161 (83.0%)	156 (75.7%)	1.10 (Lower limit 1.01)	0.05
**SECONDARY (SAFETY) OUTCOMES**				
**Early anaphylactic-type reactions**				
**Patients experiencing ≥1 reaction**	50 (25.8%)	39 (18.9%)	1.36 (Upper limit 1.86)	0.06
**Early pyrogenic reactions**	0	0	inestimable	N/A
**Late serum sickness type reactions**	5/49 (10.2%)	3/58 (5.2%)	1.97 (Upper limit 6.28)	0.27

1 Lower limit appropriate when comparing antivenom effectiveness (positive events); upper limit appropriate when comparing safety (negative events).

2 One-sided P-values calculated using Fisher's Exact test.

Recurrent blood incoagulability after initial restoration of coagulability occurred in 24 cases, 7 after ET-Plus and 17 after ET-G (Table 3). As per protocol, rescue treatment was given to patients whose blood was incoagulable 24 hr after starting antivenom treatment (Table 3). In all 400 patients, coagulopathy had resolved permanently within 60 hours of starting antivenom and there were no fatalities (Table 3).

**Table 3:** Ancillary clinical outcomes.

Outcome	EchiTAb Plus-ICP	EchiTAb G	Relative Risk^1^	P-value^2^
	(n = 194)	(n = 206)	(one-sided 95% CI)	
**Permanent restoration of blood coagulability (20WBCT) 24 hr after 1st dose, having received total of 1-4 doses**	184 (94.8%)	192 (93.2%)	1.02 (Lower limit 0.98)	0.32
				
**Recurrent blood incoagulability after initial restoration of blood coagulability**				
	7 (3.6%)	17 (8.3%)	0.44 (Upper limit 0.90)	0.04
**Patients requiring rescue treatment because their blood was incoagulable 24 hr after 1^st^ dose**				
	2 (1.0%)	2 (0.97%)	1.06 (Upper limit 5.46)	0.67
				
**Antivenom reactions**				
**Early anaphylactic-type reactions to:**				
** 1^st^ dose of antivenom**	48 (24.7%)	36 (17.5%)	1.42 (Upper limit 1.96)	0.05
** 2^nd^ dose**	4/33 (12.1%)	4/50 (8.0%)		
** 3^rd^ dose**	0/7 (0.0%)	0/9 (0.0%)		
** 4^th^ dose**	1/3 (33.3%)	0/4 (0.0%)		
**% total early reactions/total antivenom**				
**doses administered**	53/238 (22.3%)	40/270 (14.8%)		
**% severe early reactions/total antivenom**				
**doses administered**	21/238 (8.8%)	11/270 (4.1%)		
**Severity of antivenom reactions:**				
**Total patients experiencing reactions**	50 (25.8%)	39 (18.9%)	1.36 (Upper limit 1.86)	0.06
**Patients experiencing pruritis and/or urticaria only (“Mild”)**	32 (16.5%)	29 (14.1%)	1.17 (Upper limit 1.73)	0.3
**Patients experiencing bronchospasm or gastro-intestinal symptoms (“Severe”)**				
	21 (10.8%)	11 (5.3%)	2.03 (Upper limit 3.66)	0.03
**Packed cell volume (%) number**				
**<30% on discharge from hospital**	13 (6.7%)	15 (7.3%)	0.92 (Upper limit 1.68)	0.49
**Necrosis**	7 (3.6%)	15 (7.3%)	0.50 (Upper limit 1.03)	0.08
**Fatalities**	0	0	unestimable	N/A
**Length of hospital stay (hours)**				
**median (interquartile range)**	120 (96–120)	120 (96–144)	Zero (Upper limit zero)	0.2

1 Lower limit provided when comparing antivenom effectiveness (positive events); upper limit provided when comparing safety (negative events).

2 One-sided P-values calculated using Fisher's Exact test (apart from Length of hospital stay).

### Secondary (Safety) end points

1. **Anaphylactic-like reactions**
  ET-Plus caused one or more reactions on more occasions than ET-G [50/194 (25.8%) and 39/206 (18.9%) respectively [RR 1.36 one-sided 95% CI upper limit 1.86; P = 0.06]. Among the patients who reacted to ET-Plus and ET-G, 21 (10.8%) and 11 (5.3%) respectively experienced bronchospasm or gastrointestinal symptoms defined as “severe” [RR 2.03 one-sided 95% upper limit 3.66; P = 0.03] (Table 3).
2. **Pyrogenic reactions**
  No pyrogenic reactions were observed.
3. **Late serum sickness-type reactions**
  Only 107 patients (26.8%) attended for follow-up. Of the 49 of them who had received ET-Plus, 5 had late reactions and of the 58 who had received ET-G, 3 had late reactions.

#### Ancillary clinical outcomes

Spontaneous systemic bleeding was detected in 71/194 (36.6%) and 79/206 (38.3%) of patients in the ET-Plus and ET-G groups before antivenom treatment. It had ceased within 32 minutes of starting antivenom treatment in all patients and did not recur in any case. Only one patient required blood transfusion after his PCV fell to 17%. Local necrosis was not evident on admission but 7/194 of ET-Plus-treated patients (3.6%) and 15/206 of ET-G-treated patients (7.3%) developed necrosis during their hospital admission [Table 3; RR 0.50 one-sided 95% CI upper limit 1.03; P = 0.08]. Surface area of necrotic skin, and numbers requiring surgical debridement, skin grafting or amputation were not significantly different.

ET-Plus also caused severe reactions (bronchospasm/GI symptoms) on significantly more occasions than did ET-G [21/194 (10.8%) and 11/206 (5.3%) respectively [RR 2.03 one-sided 95% CI upper limit 3.66; P = 0.03]. All other early reactions were mild (pruritus or urticaria only).

Duration of hospitalization was similar in the two groups (Table 3).

## Discussion

### Effectiveness

In more than three quarters of the patients in this trial, blood coagulability was restored within 6 hr of the initial doses of ET-Plus and ET-G antivenoms, with no recurrence of incoagulability 12, 18, 24 or 48 hr later. The initial doses had been derived from preclinical test potency and confirmed by preliminary open dose-finding and safety studies [35]. Restoration of blood coagulability has been used as a surrogate marker of antivenom effectiveness in many clinical studies of viper-bite-induced consumption coagulopathy [3]-[6], [8]-[17]. In this respect, ET-Plus not only proved non-inferior to ET-G, but showed weak evidence of superiority (RR 1.10 one-sided 95% CI lower limit 1.01; P = 0.05). The low incidence of recurrent coagulopathy (7 after ET-Plus, 17 after ET-G see Table 3) contrasted with the frequency of this phenomenon when Fab fragment antivenoms are used. This is partly attributable to the slower elimination of whole IgG antivenoms [8], [30], [36]. The effectiveness of ET-Plus and ET-G compares favourably with the results published for a new candidate equine F(ab')_2_ antivenom with specificity for *E. ocellatus* venom, African Antivipmyn*®* (Laboratorios Silanes, Mexico) [12], . Among 289 patients recruited to an open multi-centre trial in Benin, West Africa, 79% had incoagulable blood suggesting systemic envenoming by *E. ocellatus* and 3% died. After treatment with an initial dose of 2 vials of African Antivipmyn*®* (based on results of pre-clinical rodent assays) that was repeated according to clinical criteria, 18% of the patients still had incoagulable blood 24 hr after starting treatment and restoration of blood coagulability was delayed beyond 60 hours in 6% of them [5]. Recurrent coagulopathy occurred in 25% of their patients compared to 6% in ours. Bleeding was arrested within 2 hours in 60% and within 24 hours in 80%, compared to an upper limit of 32 minutes in our patients. Surprisingly, results of a subsequent preliminary dose finding study of African Antivipmyn*®* in 129 patients suggested that reducing the average total dosage of antivenom from 3.81 to 2.21 vials per patient did not reduce the efficacy of the treatment, implying a rather flat dose-response curve [38].

### Safety

ET-Plus caused one or more reactions in 50/194 (25.8%) patients compared to 39/206 (18.9%) for ET-G. The incidence of these early antivenom reactions should be compared to 15.2% and 57% with 10 ml and 20 ml of the original lyophilised EchiTAb-Fab [8] and 17% with SAVP Echis antivenom [9]. In the trial of African Antivipmyn*®*, “unexpected events” were observed in 19% of patients, including shock, dyspnoea, cough and angioedema [5]. In our trial, bronchospasm or gastrointestinal symptoms, classified as severe reactions, were more frequent with ET-Plus (10.8% compared to 5.3%) probably reflecting the three times greater dose of IgG protein administered as the initial and subsequent doses of this antivenom (1.20 g) compared to ET-G (0.37 g) [35] and also possibly the greater dilution of ET-G (1∶4) compared to ET-Plus (3∶4) antivenom in the 40 ml injectate administered to all the patients. Only one quarter of the patients reported for follow up 2 weeks after the bite and among these, late serum sickness reactions were reported by 10.2% who had received ET-Plus and 5.2% ET-G (Table 2). However, these incidences may have been exaggerated if those with symptoms were more likely to return to the hospital.

There were no fatalities among the study patients but without a placebo control group the prognosis of untreated *E. ocellatus* victims fulfilling our entry criteria is uncertain. However, the danger of *E. ocellatus* envenoming is shown by the fact that, at Kaltungo Hospital, 9 *E. ocellatus* victims died after the supply of antivenom faltered in June 2009.

### Antivenoms for Africa

Throughout the African savanna north of the equator, the overwhelming need is for an antivenom to treat envenoming by Echis spp. (*E. leucogaster, E. jogeri, E. pyramidum* and especially *E. ocellatus*), the predominant cause of fatal and debilitating snake bite [1], [3], [4], [6], [8], [18]. Systemic envenoming by Echis spp. is conveniently detected by simple tests of whole blood coagulability, such as 20WBCT, allowing the use of monospecific antivenoms such as ET-G or SAVP Echis antivenom even when the causative snake cannot be identified directly. However, ET-Plus is raised against venoms of two other medically important species complexes in the same region, puff adders (*Bitis arietans*) and spitting cobras (*Naja nigricollis*), broadening its potential usefulness. Cross-neutralisation of venoms of several Echis, Bitis and Naja spp. by ET-Plus has been demonstrated in rodents [34] but its efficacy against envenoming by these species must be addressed by future clinical studies.

### Clinical testing of antivenoms

Traditionally, the clinical use of antivenoms, a neglected class of biological drugs, has been based almost entirely on results of laboratory tests in animals. In future, however, WHO [28] will strongly encourage the procedure carried out in the present programme: preclinical tests and preliminary dose-finding and safety studies [35] followed by formal Phase III clinical trials. In the present study, conventional pre-clinical rodent ED_50_ assays reliably predicted clinical effectiveness of ET-Plus and ET-G. Surely such tests should become a minimal requirement before an antivenom is selected for clinical use in a particular country and to prevent the unscrupulous marketing of geographically inappropriate antivenoms [24]. This is now the policy of the Nigerian regulatory agency NAFDAC. In the case of *E. ocellatus* bites, in which coagulopathy is a clinically relevant and objectively measurable effect of envenoming, results of our preliminary Phase I dose-finding and safety tests, using a novel 3+3 dose escalation protocol, were confirmed by the outcome of the Phase III RCT. This protocol should be considered in similar situations as a substitute for conventional Phase I studies that many consider unethical for antivenom testing [39].

### Conclusion

Comparison of initial doses of 3 vials of ET-Plus (a new antivenom) with 1 vial of ET-G (whose clinical efficacy had been established through pre-trial use and has been the standard of care since 2005) demonstrated that, at this dose, ET-Plus was slightly more effective in correcting haemostatic effects of *E. ocellatus* envenoming in Nigeria. ET-Plus has the potential advantage of a broader spectrum of activity. However, 1 vial of ET-G was slightly safer. Both antivenoms are recommended for treating *E. ocellatus* envenoming in Nigeria.

## Supporting Information

Protocol S1 Trial Protocol.
(0.23 MB DOC)

Checklist S1 CONSORT Checklist.
(0.19 MB DOC)
