# Supplementary material for: Randomised Controlled Double-Blind Non-Inferiority Trial of Two Antivenoms for Saw-Scaled or Carpet Viper (Echis ocellatus) Envenoming in Nigeria
Source: PLoS Negl Trop Dis. 2010 Jul 27;4(7):e767. doi: 10.1371/journal.pntd.0000767 (PMC2910709; doi:10.1371/journal.pntd.0000767)
Supplement: Protocol S1 — Trial Protocol. (0.23 MB DOC) [file pntd.0000767.s001.doc]

# ECHITAB STUDY GROUP NIGERIA PROTOCOL

**Phase II/III randomised-blinded-comparative-non-inferiority trial of two antivenoms for the treatment of patients envenomed by saw-scaled vipers (*Echis ocellatus*) in Kaltungo, Nigeria.**

## INTRODUCTION

Snake bite is a major medical problem in rural communities of the savanna region of West Africa, notably in Nigeria, Senegal (Trape et al., 2001), Ghana, Benin, Togo, Burkina Faso, Niger, Mali and Cameroon. Saw-­scaled vipers *(Echis ocellatus* and *E. leucogaster*) are the most important cause of snake bite mortality and morbidity in this region followed by spitting cobras (*Naja nigricollis* and *N. katiensis*) and puff adders (*Bitis arietans*). Children, farmers, herdsmen and hunters are at the greatest risk. The main clinical features of *E. ocellatus* envenoming are systemic haemorrhage, incoagulable blood, shock and local necrosis. In Nigeria, where the untreated case fatality is 10-20 per cent, this species is responsible for hundreds of deaths each year (Warrell and Arnett, 1975; Warrell *et al* 1977; Pugh and Theakston 1980; 1987; Pugh *et al* 1979). In some rural hospitals, half of the beds are occupied by snake bite victims during the peak season (e.g. during the early rainy season when farmers prepare their land for planting between May and July, and later during the harvesting period). Medical management of snake bite is extremely difficult in this area. There is a general lack of reliable data and victims of snake bite are increasingly reluctant to seek medical assistance because of the perennial shortage and expense of antivenom. The rising cost and lack of availability of antivenom in recent years has put treatment out of the reach of most patients (Theakston and Warrell, 2002; Lalloo *et al*, 2002; Laing *et al*, 2003; Theakston et al., 2003). For example, antivenom of doubtful potency may cost Naira 3,000 – 4,000 per vial from local pharmacies. As a result, useless and potentially dangerous remedies such as the black 'snake stone' have gained in popularity. Over the past ten years, this shortage of antivenom has become so dire that, at the moment, there is virtually no antivenom available in the country because traditional suppliers such as South African Vaccine Producers and Sanofi-Pasteur are unable to fulfil orders from state governments such as Gombe. As a result, increasing numbers of patients bleed to death or survive with amputated extremities.

Antivenoms previously available in Nigeria have included the South African Vaccine Producers (formerly SAIMR) and Institut Pasteur monospecific Echis antivenoms, Behringwerke and Pasteur (Bitis-Echis-Naja) polyspecific antivenoms, Pasteur Ipser Afrique, (Bitis-Echis-Naja-Dendroaspis) polyspecific antivenom and ovine monospecific *Echis ocellatus* EchiTAb (Therapeutic Antibodies Ltd [TAb] and MicroPharm Ltd). For many reasons most of these preparations are no longer available (e.g. political, cost, difficulties in production etc). More recently, a new polyspecific antivenom, FAV-Afrique (Bites, Echis, Naja, Dendroaspis) produced by Sanofi-Pasteur, has become available although supplies are extremely limited; its cost in Africa may exceed £70 (~Naira 17,500) per ampoule. Recent information from the manufacturers is that no further capital investment will be made in its production and that continuing supply is assured for only 3-5 years.

Two caprylic acid-extracted whole IgG equine antivenoms with activity against *E. ocellatus* venom have been developed by the Instituto Clodomiro Picado (Costa Rica) (EchiTAb-Plus-ICP –“ET-Plus”) and MicroPharm (London) (EchiTAb-G – “ET-G”) in collaboration with the Liverpool School of Tropical Medicine and the University of Oxford. The Costa Rican antivenom was prepared by immunising with *E. ocellatus*, *Bitis arietans* and *Naja nigricollis* venoms, selected because these are the three most important species, from a medical point of view, in sub-Saharan Africa. The MicroPharm antivenom is monospecific against *E. ocellatus* venom that has been the standard of care for *E. ocellatus* envenoming in Nigeria since 2005. Preclinical tests using WHO-approved methods showed these antivenoms to be as effective or almost as effective against *E. ocellatus* venom as the original ovine Fab fragment monospecific “EchiTAb” (Meyer et al., 1997) in doses of 3 vials (ET-Plus) and 1 vial (ET-G).

In Kaltungo Hospital, an open preliminary dose-finding and safety study in a small group of envenomed patients confirmed that these doses “cured” 3-4/6 patients while causing reactions in 0-2/6. (A third antivenom was rejected because it was too reactogenic at a dose high enough to fulfil efficacy criteria). (“Cured” meant that the patient’s blood coagulability was permanently restored 6 hours after treatment, as judged by the 20-minute whole blood clotting test). These two antivenoms will be compared in a non-inferiority RCT.

## AIMS OF THE STUDY

*Echis ocellatus* Phase II/III randomised-comparative-trial (**PII/III R-C-T**): to assess and compare the efficacy and safety (incidence and severity of acute anaphylactic reactions) of a ET-Plus, a new antivenom, with ET-G, an antivenom of established efficacy. Both antivenoms achieved adequate efficacy and safety in an open preliminary dose-finding and safety study.

## SITE OF THE STUDIES

Kaltungo General Hospital, Gombe State in Nigeria has provided an ideal setting for several previous studies of Echis bite (1972-1994) (Warrell *et al*, 1974, 1980; Daudu and Theakston, 1988; Meyer *et al*, 1997). In 1982 there were 360 admissions for snakebite with 17 deaths and in 1985 there were almost 300 bites between January and May with 76 in April alone. From January 1983 to December 1986 a total of 1578 patients were admitted to the hospital giving a mean annual admission rate of 394.5. During this period there were 59 deaths, an average of 25 deaths per year (Daudu, IJ, personal communication). More recent data (1999-2004) indicate that the incidence of snake bite, primarily due to *E. ocellatus*, is undiminished. Morbidity and mortality may be increasing as a result of the crisis in antivenom production and supply to Africa (Theakston and Warrell, 2000).

## TIME AND DURATION

Starting in June 2005 and continuing until ~400 patients have been studied (see power calculations below).

## DESIGN

**PII/III R-C-T**

Randomised, comparative, blinded study of 2 antivenoms using as the initial dose the minimum effective safe dose established in the **PI** **D-FS (preliminary Phase I dose-finding and safety study).**

***Primary end-points***

Proportion of patients given each antivenom whose blood coagulability has been permanently restored 6 hr after the first dose.

***Sample size***

The trial is designed to demonstrate non-inferiority of ET-Plus compared to ET-G. Based on pre-trial clinical use of ET-G (released for human use on humanitarian grounds because of the lack of any other available antivenom) it was estimated that permanent restoration of blood coagulability at 6 hours would be achieved in 80% of patients in the ET-G group. A 10% non-inferiority margin was deemed acceptable (i.e. at least 70% of ET-Plus patients must have permanent restoration of blood coagulability at 6 hours for it to be deemed non-inferior to ET-G). A sample size of 198 in each group provides 80% power to detect this non-inferiority margin difference of 10%, at a 5% one-sided significance level.

***Randomisation***

Patients will be allocated to receive either ET-Plus or ET-G using simple randomisation, employing a table of random numbers. Treatment allocations will be concealed in sequentially numbered opaque sealed envelopes given to the hospital pharmacist who is otherwise independent of the study. He will provide the blinded antivenoms after reconstituting them to 40mls with sterile water for injection in an unmarked syringe. The patient, clinician and outcome assessor will be blinded to the identity of the particular antivenom used.

***Statistical methods***

Baseline characteristics will be described using percentages for categorical variables, means and standard deviations for normally distributed continuous variables or medians and ranges in the case of non-normally distributed continuous variables.

To determine the magnitude and direction of the treatment effects, relative risks and one-sided 95% confidence intervals will be calculated. Continuous outcomes will be checked for normality and the treatment groups will be compared using either the *t* test (for normally distributed data) or the Mann Whitney-U test (for non-normally distributed data). Treatment effects will be presented as a corresponding difference in means or medians (plus one-sided 95% confidence intervals).

***Ethics***

ET-G and ET-Plus have been developed by the group in collaboration with the manufacturers. They will be imported and given limited registration for clinical trials by NAFDAC. The trial has been sanctioned by NAFDAC and the Gombe State Medical Research Ethics Committee. Informed consent will be obtained from each patient (or their guardian) after they have read the information sheet (in English, Hausa, Tangale and Fulani) and discussed it with medical staff.

## PATIENTS

All patients presenting to Kaltungo General Hospital, Gombe State, Nigeria (June 2005 owards) with a history of snake bite will be reviewed for possible recruitment into the study.

Inclusions: patients of both sexes and any age provided that:

1. they have incoagulable blood as defined by 20WBCT (diagnostic of systemic envenoming by *E. ocellatus* in this geographical area).
2. they have been bitten within the previous 72 hours
3. they or their relatives have given informed consent to admission, treatment and investigation

Exclusions:

1. patients who have already received antivenom for this (recent) snake bite
2. pregnant women
3. patients who are unconscious and/or have lateralising neurological signs indicative of established intracerebral haemorrhage
4. patients with a severe unrelated medical condition such as advanced AIDS or tuberculosis

Patients will be admitted to hospital and kept under observation for at least 48 hours after their blood has become coagulable because of the risk of late recurrence of incoagulable blood and bleeding.

## METHODS

**Diagnosis of the biting species:**

It will be assumed that all patients with incoagulable blood have been envenomed by *E. ocellatus*. This can be confirmed immediately in those cases where the dead snake is brought with the patient.

On admission, history and the results of physical examination will be recorded on a standard proforma (Annex I).

In particular, the following will be noted:

**Local signs:**

• Extent of local swelling (and usually of tenderness):

| **score** | Description |
| --- | --- |
| **1** | Confined to the bitten segment of limb |
| **2** | Extension to mid fore-arm of mid-calf |
| **3** | To knee or elbow |
| **4** | To mid-upper arm or thigh |
| **5** | To shoulder or inguinal ligament |
| **6** | Spreading to trunk |

• Bleeding from fang marks or local incisions

• Blistering

• Bruising

• Necrosis

• Tender, enlarged, painful lymph nodes draining the bitten area

**Systemic signs:**

• Spontaneous systemic bleeding (from gums, nose, skin, gastrointestinal or genitourinary tracts, urine etc)

• Incoagulable blood (20WBCT)

• Shock (supine systolic blood pressure less than 80 mm Hg with impaired peripheral circulation)

• Cardiac arrhythmias

**Follow-up:**

Wherever possible, patients will be encouraged to attend for follow-up assessment 2 weeks after the bite to check for late reactions (serum sickness), subsidence of local swelling, blisters and local necrosis and for any other symptoms.

**LABORATORY TESTS**

Incoagulable blood will be defined by the simple 20-minute whole blood clotting test (20 WBCT). Two ml of venous blood is placed in a new, clean, dry glass test tube and left for 20 minutes at room temperature. The tube is then tipped up to see if the blood runs out or has clotted.

Restoration of blood coagulability as estimated by the whole blood clotting test, will be taken as evidence of the efficacy of the antivenom. Persistence of incoagulable blood, measured by the 20WBCT (Sano-Martins et al, 1994), six hours after the first dose of antivenom will be an indication for repeating the initial dose.

## TREATMENT

Antivenom: The initial dose of each antivenom will be the Minimum Effective Safe Dose (MESD), as determined by the preceding PI D-FS: that is 3 vials for ET-Plus and 1 vial for ET-G. This initial dose will be repeated 6 hourly until blood coagulability is restored permanently.

### Designated “treatment failure”:

If in any patient, given either antivenom fails to achieve permanent restoration of blood coagulability within 24 hours of the first dose and subsequent further 1-3 doses (as required), the case will be designated a “treatment failure” and the patient will given “rescue treatment” with 2 vials of the “gold standard” SAVP (SAIMR) monospecific Echis antivenom.

**Recurrent envenoming:**

Once blood coagulability has been restored, 20WBCT will be repeated 6 hourly for 48 hours. If the blood becomes incoagulable again, the case will be designated “recurrent envenoming” and more trial antivenom will be given according to the previous schedule.

**General treatment:**

Early (anaphylactic) serum reactions will be treated by 1) stopping the antivenom injection) 2) giving 0.1% adrenaline intramuscularly (adult dose 0.5 - 1 ml; children 0.01 ml/kg body weight), followed by chlorphenamine maleate by slow intravenous injection (adult dose 10 mg; children 0.2 mg/kg body weight) and hydrocortisone hemisuccinate by slow intravenous injection (adult dose 100 mg; children 2 mg/kg body weight).

After the symptoms of the reaction have subsided, administration of antivenom will be completed.

Late serum sickness type reactions will be treated with chlorphenamine or prednisolone tablets.

Pain will be treated with maximum dose oral paracetamol (adult 1 g 6 hourly) or codeine phosphate (30 mg) or a stronger opioid such as pethidine, pentazocine or tramadol, depending on severity. Non-steroidal anti-inflammatory agents and aspirin will be avoided because of bleeding risks.

Fresh compatible HIV-negative whole blood will be transfused if the haematocrit falls below 20%. Envenomed limbs will be nursed in the most comfortable position. Bullae will be left alone. A booster dose of tetanus toxoid will be given to every patient. If local necrosis develops, the affected area will be surgically débrided by the resident clinician as soon as possible and broad-spectrum antimicrobial cover (flucloxacillin, gentamicin and metronidazole) started immediately.

## RECORDING OF SIDE EFFECTS OF ANTIVENOM

The following side effects will be recorded:

Within hours

- 1. Early anaphylactic reactions: generalised pruritus, urticarial rash, tachycardia, hypotension, tachypnoea, bronchospasm, angioedema, vomiting, diarrhoea, colicky abdominal pain
  2. Pyrogenic reactions: fever, chills, rigors

Within days/ 1-2 weeks

3. Late serum-sickness-type reactions: generalised pruritus, urticarial rash, arthralgias, peri-articular swellings, fever, lymphadenopathy, mononeuritis multiplex, proteinuria.

**REFERENCES**

Daudu, I.J. and Theakston, R.D.G. (1988). Preliminary testing of a new polyspecific antivenom in Nigeria. Ann. Trop. Med. Parasit., 82, 311-313.

[Gutiérrez JM, Rojas E, Quesada L, Leon G, Nunez J, Laing GD, Sasa M, Renjifo JM, Nasidi A, Warrell DA, Theakston RD, Rojas G.](http://www.ncbi.nlm.nih.gov/entrez/query.fcgi?cmd=Retrieve&db=pubmed&dopt=Abstract&list_uids=15837359&query_hl=1&itool=pubmed_docsum) Pan-African polyspecific antivenom produced by caprylic acid purification of horse IgG: an alternative to the antivenom crisis in Africa.
Trans R Soc Trop Med Hyg. (in press).

Ho, M. *et al.* (1986). A critical reappraisal of the use of ELISA in the study of snake bite. Toxicon, 24, 211-221.

Laing, G.D. et al. (2003a). Polyspecific antivenom may help in antivenom crisis. B.M.J., 326, 447-448.

Laing, G.D et al. (2003b). A new Pan African polyspecific antivenom developed in response to the antivenom crisis in Africa. Toxicon 2003 Jul;42(1):35-41.

Lalloo, D.G., Theakston, R.D.G. and Warrell, D.A. (2002). Reply to letter by Horton, R. entitled ‘Ghana: defining the African challenge’. Lancet, 359, 1527.

Malasit, P. et al. (1985) Prediction, prevention and mechanism of early (anaphylactic) antivenom reactions in victims of snake bites. B.M.J. 292, 1720.

Meyer W.P. et al. (1997) First clinical experiences with a new ovine Fab *Echis ocellatus* snake bite antivenom in Nigeria: Randomised comparative trial with Institute Pasteur serum (Ipser) Africa antivenom. Am. J. Trop. Med. Hyg. 56(3), 1997, pp. 291-300.

Pugh, R.N.H. and Theakston, R.D.G. (1980). The incidence and mortality of snake bite in savanna Nigeria. Lancet, ii, 1181-1183.

Pugh, R.N.H. et al (1977). Bites by the carpet viper in the Niger valley. Lancet, ii, 625-627.

Sano-Martins, I.S. et al. (1994). Reliability of the simple 20 minute whole blood clotting test (WBCT20) as an indicator of low plasma fibrinogen concentration in patients envenomed by Bothrops snakes. Toxicon, 32, 1045-1050.

Theakston, R.D.G. et al. (1977). Micro-ELISA for detecting and assaying snake venom and venom-antibody. Lancet, ii, 639-641.

Theakston, R.D.G. and Reid, H.A. (1993). The development of simple standard assay procedures for the characterisation of snake venoms. Bull.W.H.O., 61, 949-956.

Theakston, R.D.G. and Warrell D.A. (2000). Crisis in antivenom supply for Africa. Lancet 356, 2104.

[Theakston RD, Warrell DA, Griffiths E.](http://www.ncbi.nlm.nih.gov/entrez/query.fcgi?cmd=Retrieve&db=pubmed&dopt=Abstract&list_uids=12676433&query_hl=3&itool=pubmed_DocSum) Report of a WHO workshop on the standardization and control of antivenoms. Toxicon. 2003 Apr;41(5):541-57.

[Trape JF](http://www.ncbi.nlm.nih.gov/entrez/query.fcgi?db=pubmed&cmd=Search&itool=pubmed_Abstract&term="Trape+JF"%5BAuthor%5D), [Pison G](http://www.ncbi.nlm.nih.gov/entrez/query.fcgi?db=pubmed&cmd=Search&itool=pubmed_Abstract&term="Pison+G"%5BAuthor%5D), [Guyavarch E](http://www.ncbi.nlm.nih.gov/entrez/query.fcgi?db=pubmed&cmd=Search&itool=pubmed_Abstract&term="Guyavarch+E"%5BAuthor%5D), [Mane Y](http://www.ncbi.nlm.nih.gov/entrez/query.fcgi?db=pubmed&cmd=Search&itool=pubmed_Abstract&term="Mane+Y"%5BAuthor%5D). High mortality from snakebite in south-eastern Senegal. [Trans R Soc Trop Med Hyg.](javascript:AL_get(this, 'jour', 'Trans R Soc Trop Med Hyg.');) 2001 Jul-Aug;95(4):420-3.

Warrell, D.A. et al. (1974). Bites by the saw-scaled or carpet viper (*Echis carinatus*): a trial of two specific antivenoms. B.M.J., 4, 437-440.

Warrell, D.A. and Arnett, C. (1976). The importance of bites by the saw-scaled or carpet viper (*Echis carinatus*) in Nigeria. Quart.J.Med., 46, 33-62.

Warrell, D.A. *et al.* (1986) Comparison of Pasteur and Behringwerke antivenoms in envenoming by the carpet viper (*Echis carinatus*). B.M.J. 280, 607-609.

Warrell, D.A. et al. (1986) Randomised comparative trial of three monospecific antivenoms for bites by the Malayan pit viper (*Calloselasma rhodostoma*) in southern Thailand: Clinical and laboratory correlations. Am. J. Trop. Med. Hyg, 35, 1235-1247.

**ANNEXE 1 Clinical Proforma**

| **KALTUNGO PROJECT** |
| --- |

| STUDY NUMBER: | HOSPITAL NUMBER: |
| --- | --- |

| NAME: | | | AGE: | LANGUAGE: |
| --- | --- | --- | --- | --- |
| SEX: | M | F | WEIGHT: | HEIGHT: |

Pre existing medical conditions:

| ADDRESS: | HEAD OF  COMPOUND: | OCCUPATION: |
| --- | --- | --- |

| ADMITTED: | DATE: | TIME: |
| --- | --- | --- |
| DISCHARGED: | DATE: | DAYS IN HOSPITAL: |

| **BITTEN** | | | |
| --- | --- | --- | --- |
| DATE: | TIME: | PLACE: | PART OF  BODY: |

| SNAKE: | KILLED: | YES | NO | BROUGHT: | YES | NO |
| --- | --- | --- | --- | --- | --- | --- |

| CIRCUMSTANCES OF BITE: |
| --- |

| **SNAKE (IF NOT BROUGHT)** | | | |
| --- | --- | --- | --- |
| LENGTH: | COLOUR: | HOOD: | NAMES: |
| THICKNESS: | MARKINGS: | BEHAVIOUR: |

| **SNAKE (IF BROUGHT)** | | | | | |
| --- | --- | --- | --- | --- | --- |
| SPECIES: | SEX: | M | F | LENGTH:  BODY: mm | LENGTH:  TAIL: mm |

| **PRE-HOSPITAL TREATMENT** | | | |
| --- | --- | --- | --- |
| INCISIONS: | TOURNIQUET | | OTHER: |
|  | APPLIED: | RELEASED: |  |
|  |  |  |  |

Name: _______________________________ Study No: _________________________

| **DISPENSARY** | | |
| --- | --- | --- |
| NAME: | COMPOSITION: | HOW ADMINISTERED: |

| **NATIVE MEDICINE** | | |
| --- | --- | --- |
| NAME: | COMPOSITION: | HOW ADMINISTERED: |

| **LOCAL SYMPTOMS** | | | |
| --- | --- | --- | --- |
| SYMPTOM  MAIN COMPLAINT NOW | EXTENT  Definition? | TIME STARTED  AFTER BITE | PROGRESSION |
| PAIN |  |  |  |
| SWELLING |  |  |  |
| BLISTERING |  |  |  |
| BRUISING |  |  |  |
| BLEEDING |  |  |  |
| NECROSIS |  |  |  |
| PAINFUL NODES |  |  |  |
| OTHER |  |  |  |

| **SYSTEMIC SYMPTOMS** | | | |
| --- | --- | --- | --- |
| SYMPTOM | EXTENT | TIME STARTED AFTER BITE | PROGRESSION |
| BLEEDING GUMS |  |  |  |
| EPISTAXIS |  |  |  |
| HAEMOPTYSIS |  |  |  |
| HAEMATEMESIS |  |  |  |
| MELAENA |  |  |  |
| BLOOD IN STOOL |  |  |  |
| HAEMATURIA |  |  |  |
| BLEEDING, OTHER |  |  |  |
| SITE OF INJURY/  VENEPUNCTURE |  |  |  |
| ECCHYMOSIS |  |  |  |
| PETECHIAE |  |  |  |
| DIZZINESS |  |  |  |
| SYNCOPE |  |  |  |
| DROWSINESS |  |  |  |
| NAUSEA |  |  |  |
| VOMITING |  |  |  |
| OTHER |  |  |  |

Name: _______________________________ Study No: _________________________

| **EXAMINATION** | TIME: | DATE: |
| --- | --- | --- |

| **B I T E** | | | | | |
| --- | --- | --- | --- | --- | --- |
| SITE |  | L | R | TOOTHMARKS: | INTERVAL: mm |

**DRAWING OF BITE SITE:**

| TENDERNESS |  |
| --- | --- |
| BRUISING |  |
| NECROSIS |  |
| BLISTERING |  |
| BLEEDING |  |

| **SYSTEMIC BLEEDING** | |
| --- | --- |
| GUMS |  |
| SPUTUM |  |
| STOOL (PR) |  |
| SKIN |  |
| MUCOUS MEMBRANES |  |
| RETINAE |  |
| NOSE |  |
| VOMIT |  |
| URINE |  |

Name: _______________________________ Study No: _________________________

| **GENERAL EXAMINATION** | |
| --- | --- |
| TEMPERATURE |  |
| BLOOD PRESSURE |  |
| PULSE RATE |  |
| RHYTHM |  |
| ANAEMIA |  |
| JAUNDICE |  |
| AUSCULTATION: HEART |  |
| AUSCULTATION: LUNGS |  |
| ABDOMEN |  |

| **NEUROLOGICAL EXAMINATION** | |
| --- | --- |
| MENINGISM |  |
| CONSCIOUSNES |  |
| CRANIAL NERVES |  |
| PTOSIS |  |
| OPHTHALMOPLEGIA |  |
| PUPILS |  |
| REFLEXES |  |

| **TREATMENT** | |
| --- | --- |
| LOCAL |  |
| ANALGESIC |  |
| SEDATIVE |  |
| ANTIMICROBIAL |  |
| ANTI-TETANUS |  |
| TRANSFUSION |  |
| OTHER |  |

| **ANTIVENOM** | |
| --- | --- |
| TYPE: | ROUTE: |

20 WBCT

| DOSE | DATE | START time? | FINISH time? | **20 WBCT?** | REACTION |
| --- | --- | --- | --- | --- | --- |
| 1. |  |  |  |  |  |
| 2. |  |  |  |  |  |
| 3. |  |  |  |  |  |
| 4. |  |  |  |  |  |
| 5. |  |  |  |  |  |

Name: _______________________________ Study No: _________________________

| **CLINICAL PROGRESS** | | | | |
| --- | --- | --- | --- | --- |
| DATE |  |  |  |  |
| TIME |  |  |  |  |
| DAY POST BITE |  |  |  |  |
| NEW SYMPTOMS |  |  |  |  |

| **EXAMINATION** |
| --- |

| **B I T E** | | | | |
| --- | --- | --- | --- | --- |
| SWELLING, EXTENT |  |  |  |  |
| TENDERNESS |  |  |  |  |
| BRUISING |  |  |  |  |
| BLISTERING |  |  |  |  |
| BLEEDING |  |  |  |  |
| NECROSIS |  |  |  |  |

| **SYSTEMIC BLEEDING** | | | | |
| --- | --- | --- | --- | --- |
| GUMS/NOSE |  |  |  |  |
| SPUTUM |  |  |  |  |
| STOOL/VOMIT |  |  |  |  |
| URINE |  |  |  |  |
| SKIN/MUCOUS |  |  |  |  |
| RETINAE |  |  |  |  |

| **GENERAL** | | | | |
| --- | --- | --- | --- | --- |
| TEMPERATURE |  |  |  |  |
| ANAEMIA |  |  |  |  |
| JAUNDICE |  |  |  |  |
| BLOOD PRESSURE |  |  |  |  |
| PULSE RATE/ RHYTHM |  |  |  |  |
| AUSCULTATION |  |  |  |  |
| ABDOMEN |  |  |  |  |

| **NEUROLOGICAL** | | | | |
| --- | --- | --- | --- | --- |
| CONSCIOUSNESS |  |  |  |  |
| MENINGISM |  |  |  |  |
| CRANIAL NERVES |  |  |  |  |
| REFLEXES |  |  |  |  |

Name: _______________________________ Study No: _________________________

| **INVESTIGATIONS** | | | | | | |
| --- | --- | --- | --- | --- | --- | --- |
| DATE |  |  |  |  |  |  |
| TIME |  |  |  |  |  |  |
| DAY POST BITE |  |  |  |  |  |  |
| 20 WBCT |  |  |  |  |  |  |
| SERUM STORED |  |  |  |  |  |  |
| PCV |  |  |  |  |  |  |
| WOUND ASP |  |  |  |  |  |  |
| BLISTER ASP |  |  |  |  |  |  |
| SERUM IMM |  |  |  |  |  |  |
| URINE STIX |  |  |  |  |  |  |
|  |  |  |  |  |  |  |
|  |  |  |  |  |  |  |
|  |  |  |  |  |  |  |
|  |  |  |  |  |  |  |

**ANNEXE 2** Table of random numbers

| **TABLE OF RANDOM NUMBERS** |
| --- |
| **39634 62349 74088 65564 16379 19713 39153 69459 17986 24537** |
| **14595 35050 40469 27478 44526 67331 93365 54526 22356 93208** |
| **30734 71571 83722 79712 25775 65178 07763 82928 31131 30196** |
| **64628 89126 91254 24090 25752 03091 39411 73146 06089 15630** |
| **42831 95113 43511 42082 15140 34733 68076 18292 69486 80468** |
|  |
| **80583 70361 41047 26792 78466 03395 17635 09697 82447 31405** |
| **00209 90404 99457 72570 42194 49043 24330 14939 09865 45906** |
| **05409 20830 01911 60767 55248 79253 12317 84120 77772 50103** |
| **95836 22530 91785 80210 34361 52228 33869 94332 83868 61672** |
| **65358 70469 87149 89509 72176 18103 55169 79954 72002 20582** |
|  |
| **72249 04037 36192 40221 14918 53437 60571 40995 55006 10694** |
| **41692 40581 93050 48734 34652 41577 04631 49184 39295 81776** |
| **61885 50796 96822 82002 07973 52925 75467 86013 98072 91942** |
| **48917 48129 48624 48248 91465 54898 61220 18721 67387 66575** |
| **88378 84299 12193 03785 49314 39761 99132 28775 45276 91816** |
|  |
| **77800 25734 09801 92087 02955 12872 89848 48579 06028 13827** |
| **24028 03405 01178 06316 81916 40170 53665 87202 88638 47121** |
| **86558 84750 43994 01760 96205 27937 45416 71964 52261 30781** |
| **78545 49201 05329 14182 10971 90472 44682 39304 19819 55799** |
| **14969 64623 82780 35686 30941 14622 04126 25498 95452 63937** |
|  |
| **58697 31973 06303 94202 62287 56164 79157 98375 24558 99241** |
| **38449 46438 91579 01907 72146 05764 22400 94490 49833 09258** |
| **62134 87244 73348 80114 78490 64735 31010 66975 28652 36166** |
| **72749 13347 65030 26128 49067 27904 49953 74674 94617 13317** |
| **81638 36566 42709 33717 59943 12027 46547 61303 46699 76243** |
|  |
| **46574 79670 10342 89543 75030 23428 29541 32501 89422 87474** |
| **11873 57196 32209 67663 07990 12288 59245 83638 23642 61715** |
| **13862 72778 09949 23096 01791 19472 14634 31690 36602 62943** |
| **08312 27886 82321 28666 72998 22514 51054 22940 31842 54245** |
| **11071 44430 94664 91294 35163 05494 32882 23904 41340 61185** |
|  |
| **82509 11842 86963 50307 07510 32545 90717 46856 86079 13769** |
| **07426 67341 80314 58910 93948 85738 69444 09370 58194 28207** |
| **57696 25592 91221 95386 15857 84645 89659 80535 93233 82798** |
| **08074 89810 48521 90740 02687 83117 74920 25954 99629 78978** |
| **20128 53721 01518 40699 20849 04710 38989 91322 56057 58573** |
|  |
| **00190 27157 83208 79446 92987 61357 38752 55424 94518 45205** |
| **23798 55425 32454 34611 39605 39981 74691 40836 30812 38563** |
| **85306 57995 68222 39055 43890 36956 84861 63624 04961 55439** |
| **99719 36036 74274 53901 34643 06157 89500 57514 93977 42403** |
| **95970 81452 48873 00784 58347 40269 11880 43395 28249 38743** |
|  |
| **56651 91460 92462 98566 72062 18556 55052 47614 80044 60015** |
| **71499 80220 35750 67337 47556 55272 55249 79100 34014 17037** |
| **66660 78443 47545 70736 65419 77489 70831 73237 14970 23129** |
| **35483 84563 79956 88618 54619 24853 59783 47537 88822 47227** |
| **09262 25041 57862 19203 86103 02800 23198 70639 43757 52064** |
